# Shouhui Tongbian Capsule ameliorates 5-fluorouracil induced constipation in mice by modulating gut microbiota and activating PI3K/AKT/AQP3 signaling pathway

**DOI:** 10.3389/fmicb.2025.1596881

**Published:** 2025-07-10

**Authors:** Yawei Zhang, Yang Dong, Chenghong Sun, Lufan Zhang, Yi Zhang, Dan Wang, Qian Chen, Jingchun Yao, Yuzheng Wu, Tao Wang

**Affiliations:** ^1^State Key Laboratory of Chinese Medicine Modernization, Tianjin University of Traditional Chinese Medicine, Tianjin, China; ^2^State Key Laboratory of Component-based Chinese Medicine, Tianjin University of Traditional Chinese Medicine, Tianjin, China; ^3^State Key Laboratory of Integration and Innovation of Classic Formula and Modern Chinese Medicine, Lunan Pharmaceutical Group Co. Ltd, Linyi, China; ^4^Institute of Traditional Chinese Medicine, Tianjin University of Traditional Chinese Medicine, Tianjin, China

**Keywords:** Shouhui Tongbian Capsule (SHTC), 5-fluorouracil (5-FU), chemotherapy-induced constipation (CIC), gut microbiota, fecal microbiota transplantation (FMT)

## Abstract

**Objective:**

Shouhui Tongbian Capsule (SHTC) has been clinically applied to treat various types of constipation, including chemotherapy-induced constipation. However, the pharmacological mechanism by which it regulates intestinal peristalsis and treats constipation is unclear. In this study, we aimed to investigate the underlying mechanism of SHTC on chemotherapy-induced constipation through regulating of gut microbiota and PI3K/AKT/AQP3 signaling pathway.

**Methods:**

Chemotherapy-induced constipation was induced with 5-Fluorouracil in C57BL/6 mice. SHTC was administrated with different dosages (100, 200, 400 mg/kg) for 12 days. The intestinal tissues were collected for the measurements of intestinal propulsion rate, time of first black stool, and expressions of colonic aquaporin. 16S rRNA sequencing, short-chain fatty acids (SCFAs) profiling, and fecal microbiota transplantation (FMT) were performed to confirm whether gut microbiota is a key target for SHTC. Finally, the expressions of proteins or genes related to PI3K/AKT/AQP3 pathway were detected.

**Results:**

SHTC markedly improved the pathological manifestations associated with constipation and restored the deregulated gut microbiota. The mice that were given fecal supernatant from SHTC-treated mice showed significant improvement in constipation symptoms. Additionally, SHTC increased the level of acetic acid and upregulated the expression of AQP3, with activation of PI3K/AKT. Furthermore, the blockade of PI3K reversed the beneficial effect of acetic acid on the expression of AQP3.

**Conclusion:**

Our findings indicated that SHTC effectively relieved 5-FU-induced constipation in mice, mainly by regulating homeostasis of gut microbiota and activating PI3K/AKT/AQP3 pathway, making it a potential protective agent against chemotherapy-induced constipation.

## 1 Introduction

Constipation is one of the most prevalent gastrointestinal problems in cancer patients, characterized by infrequent and hard stools, excessive straining, and a sense of anorectal blockage (Dzierżanowski and Mercadante, 2022). Constipation not only brings distress to patients and affects their quality of life, but also decreases compliance with chemotherapy, which deteriorates the overall performance of treatment (Davies et al., 2020). Therefore, some adjuvant drugs should be considered to relieve the constipation caused by chemotherapy, to soothe the discomfort of patients during chemotherapy, and to improve the therapeutic effect.

At present, the methods clinically used to alleviate chemotherapy-induced constipation include lifestyle changes and the use of medications (Bellini et al., 2021; Włodarczyk et al., 2021). The medications commonly used to improve intestinal dysmotility after chemotherapy include laxatives, pro-secretory agents, serotonergic agonists, probiotics, and prebiotics (Włodarczyk et al., 2021). However, despite the therapeutic benefits of these medications, chemotherapy-induced constipation remains a major problem for cancer patients, since these drugs can cause a variety of side effects, such as headache, nausea, and electrolyte disorders (Bharucha and Wald, 2019). Therefore, to relieve these discomforts of the gastrointestinal tract, more and more people are now choosing traditional Chinese medicine (TCM) as a complementary treatment.

Shouhui Tongbian Capsule (SHTC) is a Chinese patent medicine clinically used to treat functional constipation. Recent pharmacological studies have shown that SHTC can also improve obesity and metabolic disorders (Wu et al., 2025), and protect against cerebral ischemic stroke (Wei et al., 2025). The whole formula of SHTC consists of 8 traditional Chinese herbs, namely *Fallopia multiflora* (Thunb.) Harald, *Aloe barbadensis* Miller, Cassiae Semen (*Cassia obtusifolia* L.), Wolfberry (*Lycium barbarum* L.), Corii Colla Asini (*Equus asinus* L.), *Panax ginseng* C. A. Meyer, *Atractylodes macrocephala* Koidz, and *Citrus aurantium* L. In accordance with TCM theory, *Panax ginseng* C. A. Meyer and *Aloe barbadensis* Miller exert therapeutic effects of tonifying Qi, clearing liver heat, and increasing fluid production in the colon. *Fallopia multiflora* (Thunb.) Harald and Corii Colla Asini (*Equus asinus* L.) demonstrate pharmacological actions of nourishing Yin and lubricating intestinal tract. Cassiae Semen (*Cassia obtusifolia* L.), Wolfberry (*Lycium barbarum* L.), *Atractylodes macrocephala* Koidz, and *Citrus aurantium* L. collectively manifest therapeutic properties including heat purgation, defecation promotion, Qi supplementation, Yin nourishment, and intestinal lubrication (Gong et al., 2022). These herbal medicines exhibit synergistic interactions that collectively mediate therapeutic effects in constipation management. Several contemporary pharmacological studies have reported the mechanism of SHTC in improving constipation symptoms. For example, Sun et al. have reported that SHTC can effectively regulate energy metabolism in the colon, including tyrosine and tryptophan biosynthesis, arginine biosynthesis, glycolysis, and tricarboxylic acid cycle, which may indirectly improve constipation symptoms (Sun et al., 2021). Bai J et al. have reported that SHTC significantly ameliorated loperamide-induced experimental constipation and accelerated enteric motility *via* promoting 5-HT biosynthesis in enterochromaffin cells, as well as promoted enteric neuron growth of the enteric nervous system (ENS) in both the small intestine and colon (Bai et al., 2022). In addition, SHTC was found to improve intestinal motility in mice with loperamide-induced constipation by increasing the relative abundance of *Lactobacillus*, increasing the ratio of *Firmicutes* to *Bacteroides* (F/B), and up-regulating the levels of acetic acid and propionic acid (Lin et al., 2022). However, whether SHTC has a therapeutic effect on chemotherapy-induced constipation has not been investigated.

In the present study, we aimed to confirm the therapeutic effect of SHTC on chemotherapy-induced intestinal dysmotility in mice with 5-FU-induced experimental constipation, followed by attempting to demonstrate the underlined mechanism in terms of the therapeutic effects of SHTC on intestinal dysmotility.

## 2 Materials and methods

### 2.1 Reagents

Shouhui Tongbian Capsule (SHTC) was produced by Lunan Pharmaceutical Group Co., Ltd. (Linyi, China, manufacture batch number: 26210523). SHTC is composed of 120 g *Polygonum multiflorum* Thunb, 160 g *Aloe barbadensis* Miller, 140 g Cassiae Semen (*Cassia obtusifolia* L.), 75 g Wolfberry (*Lycium barbarum* L.), 75 g Corii Colla Asini (*Equus asinus* L.), 50 g *Panax ginseng* C. A. Meyer, 50 g *Atractylodes macrocephala* Koidz, 120 g *Citrus aurantium* L. Moxapride citrate tablets were purchased from Jiangsu Haosen Pharmaceutical Group Co., Ltd. (Jiangsu, China). 5-fluorouracil (5-FU, B25419) was purchased from Shanghai Yuanye Biotechnology Co., Ltd. (Shanghai, China). Activated carbon (C7261), gum Arabic (G8130), and Trizol (Lot#Q30704) were obtained from Beijing TransGen Biotechnology Co., Ltd. (Beijing, China). SYBR Green PCR Master Mix (CW2623) and High Capacity cDNA Reverse Transcription kit (CW2569) were obtained from Applied Biosystems (Waltham, MA, USA). Mouse Gastric Actin ELISA Kit (MTL, JL10463), Mouse Acetylcholinesterase ELISA Kit (AChE, JL20661), Mouse Endothelin 1 ELISA Kit (ET-1, JL12801) were purchased from Jiangsu Jianglai Biotechnology Co., Ltd. (Nanjing, China). LY294002 was purchased from MedChemExpress (New Jersey, USA) and sodium acetate was purchased from MACKLIN (Shanghai, China).

### 2.2 UPLC analysis

The UPLC method was referenced from previous literature with minor modifications (Liang et al., 2022; Ma et al., 2022). 0.5 g SHTC was added with 50 mL 50% methanol and sonicated in an ultrasonic water bath for 60 min. The supernatant was collected and filtered through a 0.22 μm filter membrane for further analysis. The concentrations of 2,3,5,4′-tetrahydroxyl diphenylethylene-2-o-glucoside, Naringin, and Aloin in the extracts of SHTC were analyzed using the Waters ACQUITY UPLC H-class system (Waters Corporation, Milford, MA) and C18 column (Waters ACQUITYTM UPLC BEH C18 column, 2.1 mm × 100 mm, 1.7 μm). The following settings were used for UPLC analysis: mobile phase, 0.05% phosphoric acid aqueous solution (A); acetonitrile (B) at an elution flow rate of 0.3 mL/min; column temperature 30°C. Gradient elution with A (0–5.0 min, 85–80% A; 5.0–7.0 min, 80% A; 7.0–10.0 min, 80–85% A; 10.0–12.0 min, 85% A). The detection wavelength is 220 nm.

### 2.3 Animal experiment

C57BL/6 male mice (20–22 g) were purchased from Beijing Spelford Biotechnology Co. Ltd. and housed under standard conditions (20 ± 2°C, 50–60% humidity, and 12 h light/dark cycle) in the Experimental Animal Center of Tianjin University of Chinese Medicine. The mice were allowed free access to mouse chow (components: soybean meal, fish meal, vegetable oil, bran, maize, wheat middlings, vitamins, minerals, etc.; purchased from Beijing Huafukang Biotechnology Co., Ltd., batch number: 1022), and the drinking water was purified water sterilized by high pressure. All animal studies were approved by the Science and Technological Committee and the Animal Use and Care Committee of Tianjin University of Traditional Chinese Medicine (TCM-LAEC2022205).

### 2.4 5-FU induced constipation in mice

**Figure 1:**
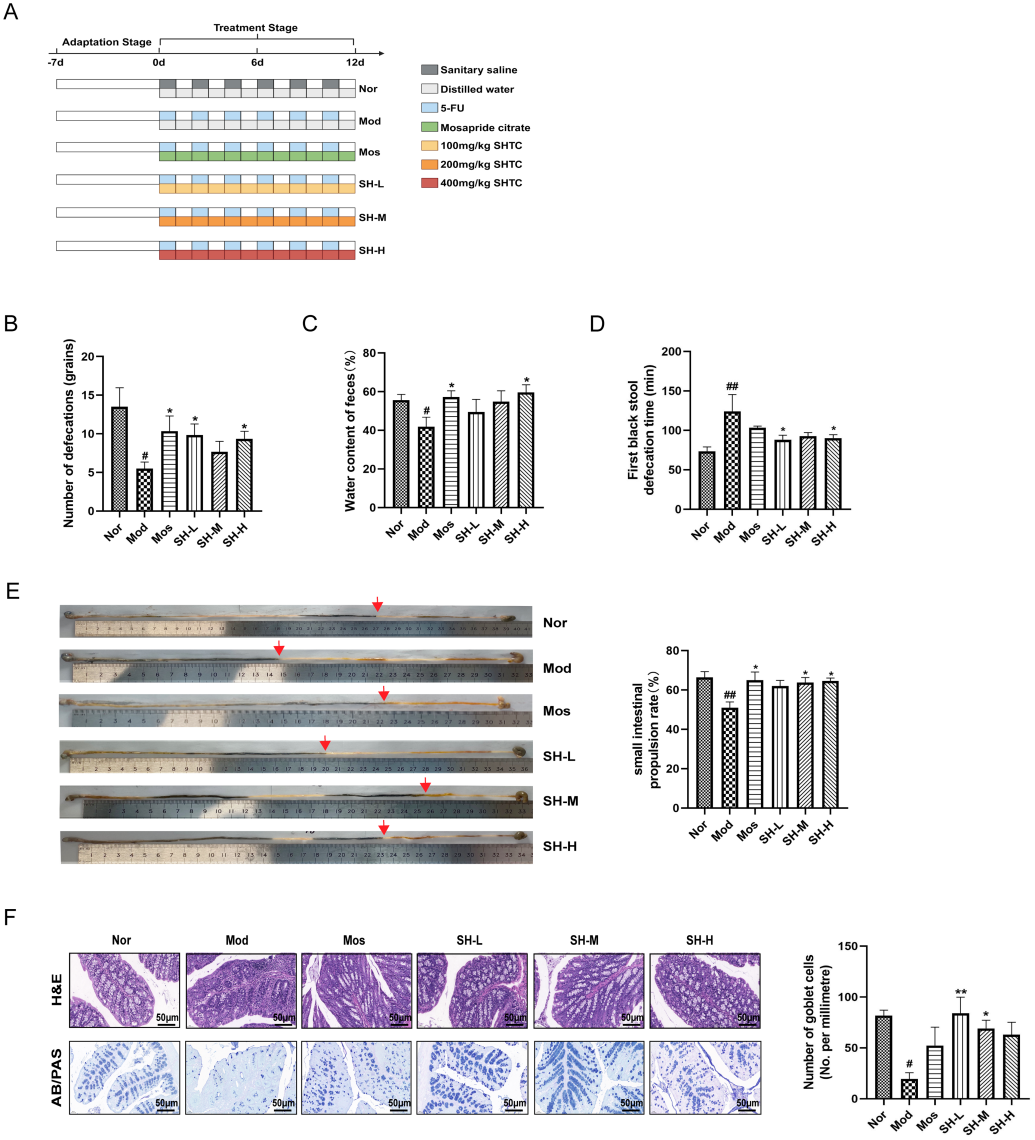
SHTC relieves constipation symptoms in mice. **(A)** Experimental design; **(B)** Number of bowel movements; **(C)** Water content of feces; **(D)** Time to first black stool; **(E)** small intestinal propulsion in mice. **(F)** H&E and AB/PAS staining of colonic tissue. Data are reported as mean ± SEM (*n* = 6/8) and analyzed using One-way ANOVA. ^#^*p* < 0.05, ^##^*p* < 0.01 vs. Normal group; ^*^p < 0.05, ^**^p < 0.01, vs. Model group.

After acclimation for 1 week, the mice were randomly divided into normal group (Nor), model group (Mod), Mosapride Citrate group (Mos), SHTC low dose group (SH-L), SHTC medium dose group (SH-M), SHTC high dose group (SH-H), with 8 mice per group. All the mice except the ones in the control group, were injected intraperitoneally with 46 mg/kg 5-FU [the administered dose of 5-FU was determined according to the report of Justino et al. (2015) and McQuade et al. (2019) and the pre-experiment] on the first day of the experiment. Then every other day for 12 days for a total of 6 injections, the mice in the normal group were injected with the same dose of saline. SHTC or mosapride citrate was given 1 h after intraperitoneal injection of 5-FU. The mice in the Mos group and SH-L/M/H groups were orally administered Mosapride Citrate (20 mg/kg) or SHTC (100, 200, 400 mg/kg). All treatments were administered once daily for 12 consecutive days. The doses of Mosapride Citrate and SHTC were determined according to the eport of Suchitra et al. (2003) and Bai et al. (2022). The mice in the Nor group and Mod group were given the same volume of saline. On the tenth and twelfth days of the experiment, all mice were fasted for 16 h and gavaged with carbon powder suspension to detect the time of the first black stool and the rate of small intestinal propulsion. The experimental design is shown in Figure 1A.

Preparation of carbon powder suspension: The preparation method was referenced from published literature with minor modifications (Lin C. Y. et al., 2015). Accurately weigh 100 g of gum arabic and add it to 800 mL of water. Boil the solution until it becomes transparent, then add 50 g of activated carbon and boil the solution three times. After the solution cools, dilute it to 1,000 mL with water, store it at 4°C, and mix well before use.

### 2.5 Fecal microbiota transplantation (FMT)

Sixty mice were randomly divided into the donor group (*n* = 30) and the recipient group (*n* = 30). Among them, the donor mice were randomly divided into Nor group (*n* = 10), 5-FU group (*n* = 10), and 5-FU + SH group (*n* = 10). All donor mice except those in the Nor group were intraperitoneally injected with 46 mg/kg of 5-FU on the first day of the experiment, followed by intraperitoneal injections every other day, and mice in the 5-FU + SH group were gavaged with 400 mg/kg of SHTC every day for 8 days. The recipient mice were randomly divided into FMT-Nor group (*n* = 10), FMT-Mod group (*n* = 10) and FMT-SH group (*n* = 10). All recipient mice were gavaged with 0.2 mL of the antibiotic mixture, referred to as ABX (ampicillin 100 mg/kg; metronidazole 100 mg/kg; neomycin 100 mg/kg; and vancomycin 50 mg/kg) daily for 7 days prior to the start of the experiment. After 2 days of intestinal rest, all recipient mice were intraperitoneally injected with 46 mg/kg of 5-FU every other day to induce constipation. Meanwhile, each group of recipient mice received freshly prepared fecal supernatant (200 μL/20 g) from the corresponding donor mice by gavage for 8 days. On the sixth day of administration, all recipient mice were fasted for 16 h. After administration of fecal supernatant for 30 min, carbon powder suspension was gavaged and the time to the first black stool, 4-h fecal excretion and fecal water content was measured. On the eighth day of the experiment, all the recipient mice were fasted for 16 h, given fecal supernatant for 30 min, and then gavaged with carbon powder suspension. Twenty minutes later, all mice were sacrificed, the rate of small intestine propulsion was calculated, and mouse serum and colon tissue were collected for subsequent experiments.

Fecal supernatant preparation: This method was referenced from published literature (Gray et al., 2024). Feces from each group of donor mice are collected daily, diluted with PBS (100 mg/mL), vigorously vortexed to form a homogeneous suspension. The suspension is centrifuged at 600 × g for 10 min at 4°C, and the supernatant is collected for subsequent use.

### 2.6 Evaluation of constipation symptoms

Mice were given 10 ml/kg activated carbon suspension at the concentration of 5%, and then the mice were immediately placed into metabolic cages and the time of the first black stool was started to be recorded, and the feces were collected for 4 h. The feces were weighed before and after they were dried at 60°C in a drying oven. The water content of the feces was calculated according to the following formula: (wet weight of feces- dry weight of feces)/wet weight of feces× 100%.

The intestinal propulsion rate was measured according to the method reported by Hao M et al. with minor modifications (Hao et al., 2023). At the end of the animal experiment, the mice were fasted overnight for 16 h to empty the intestinal contents. On the day of sampling, 10 ml/kg of 5% activated carbon suspension was given to the mice orally. After 20 min, the abdominal cavity of each mouse was opened rapidly, the intestine from the pylorus to the ileocecal valve was collected, and the whole length of the small intestine was measured and recorded. The small intestinal propulsive rates were calculated according to the following formula: Small intestine propulsive rate (%) = (black semi-solid paste front distance/whole length of the small intestine) × 100%.

The levels of excitatory gastrointestinal hormones acetylcholine (AChE) and gastrin (MTL) and inhibitory gastrointestinal hormone endothelin1 (ET-1) in mouse serum were measured using ELISA kits according to the manufacturer’s instructions. Briefly, blood was collected from the orbital sinus of the mice, and centrifuged at 3500 rpm for 15 min at 4°C after keeping it at room temperature for 2 h. The serum was collected and added to the 96-well plate which was pre-coated with the corresponding antibody. After washing and incubation with the second antibody, the concentrations of AChE, MTL, and ET-1 were detected with a microplate reader.

### 2.7 Hematoxylin–eosin (H&E) staining and AB-PAS staining

The colon tissue near the cecum was collected by 1 centimeter and fixed in 4% paraformaldehyde for 24 h. Then the tissue was embedded with paraffin, cut into 5-μm-thick slices, and stained with hematoxylin–eosin (H&E) and Alcian Blue Periodic acid Schiff (AB-PAS). Both H&E and AB-PAS sections were scanned with a light microscope for histopathological assessment.

### 2.8 Determination of mRNA levels of aquaporins and inflammatory factors

Total RNA was extracted from colon tissue using Trizol reagent. The absorbance ratios at 260 nm and 280 nm were measured by a microspectrophotometer (TECAN Corporation) to determine the purity of RNA. The concentration of RNA was measured and quantified at 2.5 μg. cDNA was synthesized with High-Capacity cDNA Reverse Transcription Kits according to the manufacturer’s instructions (Incubation at 42°C for 35 min, 85°C for 5 min). RT-PCR reactions were performed using the Applied Biosystems 7,500 Real-time PCR System (Applied Biosystems, USA), Power SYBR GREEN PCR MASTER MIX, cDNA template as well as the relative primers under the following conditions: pre-denaturation at 95°C for 10 min, denaturation at 95°C for 15 s, annealing at 60°C for 60 s, and extension at 60°C for 60 s, for a total of 40 cycles. The relative expressions of each gene were calculated according to the 2^-ΔΔCT^ method. The sample size was 6 and each sample was repeated three times. GAPDH was applied as the internal control. The sequences of the primers are displayed in Supplementary Table S1.

### 2.9 Western blot analysis

Total proteins extracted from colon tissues were quantified with a BCA protein assay kit (Thermo Fisher Scientific, Waltham, MA), then separated by sodium dodecyl sulfate-polyacrylamide gel electrophoresis (SDS-PAGE; 80 V, 35 min, 110 V, 80 min) and transferred to polyvinylidene fluoride membranes (Millipore). The membranes were incubated overnight at 4°C with the following primary antibodies: AQP3 (1:1000, ABclonal), *β*-actin (1:10000, ABclonal), PI3K (1:1000, Affinity), p-PI3K (1:1000, Affinity), AKT (1:1000, Affinity), p-AKT (1:1000, Affinity). After washing the membranes three times with Tris-buffered saline with Tween 20 (TBST, Solarbio), the membranes were incubated with the secondary antibodies for 1 h at room temperature, and then the membranes were washed three times with TBST. Finally, the bands were monitored with a chemiluminescence detection kit (Millipore), and visualized with a gel imaging analysis system (Bio-Rad). The intensity of the protein blots was calculated using ImageJ_v1.8.0.

### 2.10 Gut microbiota analysis by 16S rRNA gene sequencing

Fecal samples were collected from each mouse at the time of defecation. Microbial DNA was extracted using the CTAB method and quantified by a Nano-Photometer® NP80 (Implen, Munich, Germany). The v3-v4 region of the bacterial 16S rRNA gene was amplified with the forward primer 341F (CCTAYGGGRBGCASCAG) and reverse primer 806R (GGACTACNNGGGTATCTAAT). DNA libraries were constructed using the Tru-Seq® DNA PCR-Free Sample Preparation Kit (Illumina, USA) according to the manufacturer’s instructions. After assessing library quality with a Qubit@ 2.0 Fluorometer (Thermo Scientific) and Agilent Bioanalyzer 2,100 system, sequencing was performed on an Illumina NovaSeq platform to generate 250 bp paired-end reads. Following filtration of the sequencing data according to the QIIME V1.9.1 quality control pipeline, the data were clustered into operational taxonomic units (OTUs) at a 97% identity level using Uparse software V7.0.1001. Differences in community structure between groups were analyzed by PCoA. Based on the results of species annotation, the top 10 species with the highest genus-level abundance were selected for each sample and a cumulative bar chart of species relative abundance was generated to show the species with high genus-level relative abundance and their proportions for each sample. In addition, Alpha diversity of ASVs was performed with Shannon and Simpson to analyze the diversity, richness, and evenness of the sampled communities. To identify a differential representative of taxa and functional modules before and after SHTC treatment, a linear discriminant analysis effect size (LEfSe) analysis was used to identify differentially abundant taxa modules [those with linear discriminant analysis (LDA) score >4.0].

### 2.11 Determination of SCFAs in feces

Take 50 mg of fecal samples, homogenize and mix them with 400 μL of methanol (80%), and centrifuge to obtain the supernatant. The supernatant was added to derivatization reagent (150 μL) and derivatized at 40°C for 40 min. Then supernatant (125 μL) was homogenized with 875 μL mixed internal standard solution. Finally, injected into the LC–MS/MS system for analysis. An ultra-high performance liquid chromatography coupled to tandem mass spectrometry (UHPLC–MS/MS) system (Vanquish™ Flex UHPLC-TSQ Altis™, Thermo Scientific Corp., Germany) was used to quantitate SCFA. Separation was performed on a Waters ACQUITY UPLC BEH C18 column (2.1 × 100 mm, 1.7 μm) which was maintained at 40°C. The mobile phase, consisting of 10 mM ammonium acetate in water (solvent A) and acetonitrile: isopropanol (1:1) (solvent B), was delivered at a flow rate of 0.30 mL/min. The solvent gradient was set as follows: initial 25% B, 2.5 min; 25–30% B, 3 min; 30–35% B, 3.5 min; 35–38% B, 4 min; 38–40% B, 4.5 min; 40–45% B, 5 min; 45–50% B, 5.5 min; 50–55% B, 6.5 min; 55–58% B, 7 min; 58–70% B, 7.5 min; 70–100% B, 7.8 min; 100–25% B, 10.1 min; 25% B, 12 min. The mass spectrometer was operated in negative multiple reaction mode (MRM). Parameters were as follows: IonSpray Voltage (−4,500 V), Sheath Gas (35 psi), Ion Source Temp (550°C), Auxiliary Gas (50 psi), Collision Gas (55 psi). To further explore the relationship between the abundance of the enteric bacteria and the SCFAs, we used Pearson correlation analysis to elucidate the relationship between differential species in LEfSe and SCFAs.

### 2.12 Cell cultivation

IEC-6 cells (Cat No. FH0396) are rat small intestine crypt epithelial cells, provided by Shanghai Fuheng Biotechnology Co., Ltd. (Shanghai, China). IEC-6 cells were cultured in petri dishes with a complete medium consisting of DMEM, 10% fetal bovine serum, and 1% double-antibody. The cells were divided into four groups, namely normal group (Nor), sodium acetate-treated group (NaAc), PI3K inhibitor-treated group (LY294002), and sodium acetate + PI3K inhibitor-treated group (LY294002 + NaAc). When the cell density was about 80%, sodium acetate (NaAc, 5 mM) or PI3K inhibitor (LY294002, 20 μM) were added into the medium of corresponding groups, respectively. After incubation for 24 h, all the cells were collected for Western blot analysis.

### 2.13 Statistical analysis

The statistical analysis was performed using SPSS 25.0 statistical software (Version 25, SPSS; IBM, Armonk, NY) and GraphPad Prism 9.0 software. Data were presented as the mean ± SEM. The Shapiro–Wilk test assessed normal distribution of variables. Differences between three or more groups were evaluated using one-way ANOVA analysis, and the LSD and Dunnett’s tests were used for *post hoc* evaluations. Independent t-tests were used to compare two independent samples. Spearman’s rank correlation analysis was applied to assess the correlations between environmental factors and gut microbiota, as well as the correlations between gut microbiota abundances and SCFAs levels. *p* < 0.05 was considered statistically significant (In the figures, #, ## and ### indicate comparisons between the Model group and the Normal group/FMT-Normal group: ^#^*p* < 0.05, ^##^*p* < 0.01, ^###^*p* < 0.001; *, ** and *** indicate comparisons between each administration group and the Model group/FMT-Model group: ^*^p < 0.05, ^**^p < 0.01, ^***^p < 0.001).

## 3 Results

### 3.1 Analysis of the main components of SHTC

The concentrations of 2,3,5,4′-tetrahydroxyl diphenylethylene-2-o-glucoside, Naringin, and Aloin were analyzed using UPLC. The representative chromatograms of the mixed three standards and SHTC extracted solution are shown in Supplementary Figure S1. The concentrations of the components are as follows: 11.28 mg/g for 2,3,5,4′-tetrahydroxyl diphenylethylene-2-o-glucoside, 24.7 mg/g for Naringin, 58.74 mg/g for Aloin in the SHTC (Supplementary Table S2).

### 3.2 Observation of the fecal character of mice

The fecal characters were important symptoms for evaluating the successful establishment of a constipation model. Therefore, during the establishment of 5-FU induced constipation mice model, we observed and recorded the size, amount, shape and texture of the fecal (Supplementary Table S3). Mice in the normal group had smooth, oval shaped feces with a slightly moist surface. After the second dose of 5-FU, the mice in the model group exhibited diarrhoea-like symptoms, as evidenced by an increase in the frequency of defecation and unformed, watery stools. As the dosage of 5-FU increased, mice in the model group gradually showed signs of constipation, such as difficulty in defecation, reduced frequency of excretion, dryness and reduced volume of feces. These observations demonstrated that 5-FU could induce constipation in mice under our experimental scheme.

### 3.3 SHTC improves 5-FU induced constipation in mice

To assess the role of SHTC in 5-FU-induced constipation, three different dosages (100, 200, 400 mg/kg) of SHTC were given to the mice, and the body weight, food, and water intake were recorded. The experimental design is shown in Figure 1A. SHTC slightly restored the body weight of mice, but has no effect on food and water consumption (Supplementary Figure S2). The number of defecations, fecal water content, and defecation time were key indicators in the assessment of constipation. As shown in Figures 1B–D, 5-FU treatment significantly reduced the number of defecation and fecal water content, and increased the time of the first black stool defecation, while SHTC markedly improved these symptoms. Furthermore, SHTC treatment significantly improved 5-FU-induced inhibition of small intestine peristalsis and increased gastrointestinal propulsion rate (Figure 1E). Histological changes were observed by HE and AB/PAS staining of mouse colon tissues (Figure 1F). The colon tissues from the normal group showed healthy and integrated structures with abundant goblet cells and smooth crypt surfaces. The colon tissues from the model group showed severe loss of goblet cells. However, these histological damages were found to be significantly improved after SHTC administration. These results indicated that SHTC has the potential to improve 5-FU-induced constipation.

### 3.4 Effect of SHTC on intestinal motility regulatory biomarkers, inflammatory factors and aquaporins

**Figure 2:**
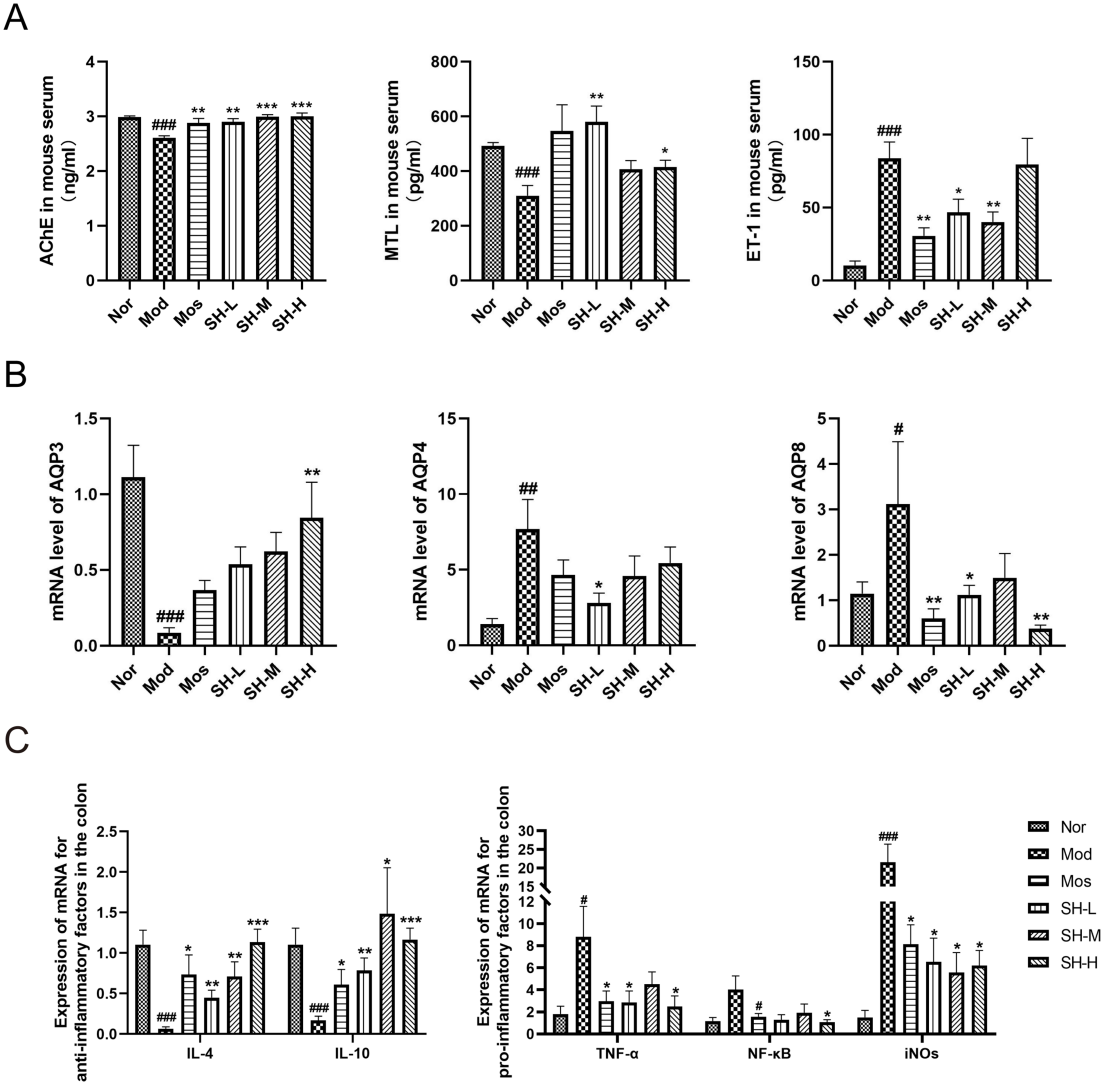
SHTC alters the levels of gastrointestinal regulation-related peptides and mRNA levels of inflammatory factors and aquaporins in constipated mice. **(A)** Serum levels of AChE, MTL, and ET-1; **(B)** mRNA levels of Aquaporins *Aqp3*, *Aqp4*, and *Aqp8* in the colon tissue of mice; **(C)** mRNA expressions of *Il-4*, *Il-10*, *Tnf-α*, *Nf-κb*, and *iNOS* in the colon tissue of mice. Data are reported as mean ± SEM (*n* = 6/8) and analyzed using One-way ANOVA. ^#^*p* < 0.05, ^##^*p* < 0.01, ^###^*p* < 0.001 vs. Normal group; ^*^p < 0.05, ^**^p < 0.01, ^***^p < 0.001 vs. Model group.

Studies have reported that AChE, MTL and ET-1 were important gastrointestinal regulation-related peptides involved in the regulation of gastrointestinal motility (Zhang et al., 2021). In this study, the results of ELISA analysis showed that the serum levels of AChE and MTL in the mice of the model group were significantly lower than those in the normal group. SHTC treatment obviously recovered the decrease of AChE and MTL (Figure 2A). The levels of ET-1 were significantly increased in the constipation model mice compared with the normal mice. Nevertheless, this alteration was reversed after SHTC administration (Figure 2A).

Aquaporins (AQPs) are a family of transmembrane proteins expressed in the gastrointestinal tract mainly responsible for water transport. The abnormal expression of AQPs is closely related to the occurrence of gastrointestinal diseases including constipation (Chu and Cai, 2023). In this study, we have observed that the mRNA levels of *Aqp3* in the colon of constipated mice were lower than those of normal mice, while the mRNA levels of *Aqp4* and *Aqp8* in the colon of constipated mice were higher than those of normal mice. SHTC administration significantly increased the expressions of *Aqp3* and decreased the expressions of *Aqp4* and *Aqp8* in the colon of constipated mice (Figure 2B).

To explore the effect of SHTC on the expressions of inflammatory factors, the levels of several cytokines were detected. As shown in Figure 2C, compared with the normal group, the mRNA expression levels of anti-inflammatory factors, *Il-4* and *Il-10*, were significantly decreased in the model group, while the expression levels of *Tnf-α*, *Nf-κb*, and *iNOS* were all significantly increased. After administration of SHTC, the levels of *Il-4* and *Il-10* were increased, *Tnf-α*, *Nf-κb*, and *iNOS* were decreased. These results suggest that SHTC can affect the levels of intestinal motility regulatory factors, modulate intestinal inflammation, control the expression of AQPs, and alleviate constipation symptoms.

### 3.5 Effect of SHTC on the composition of intestinal microbes in constipated mice

**Figure 3:**
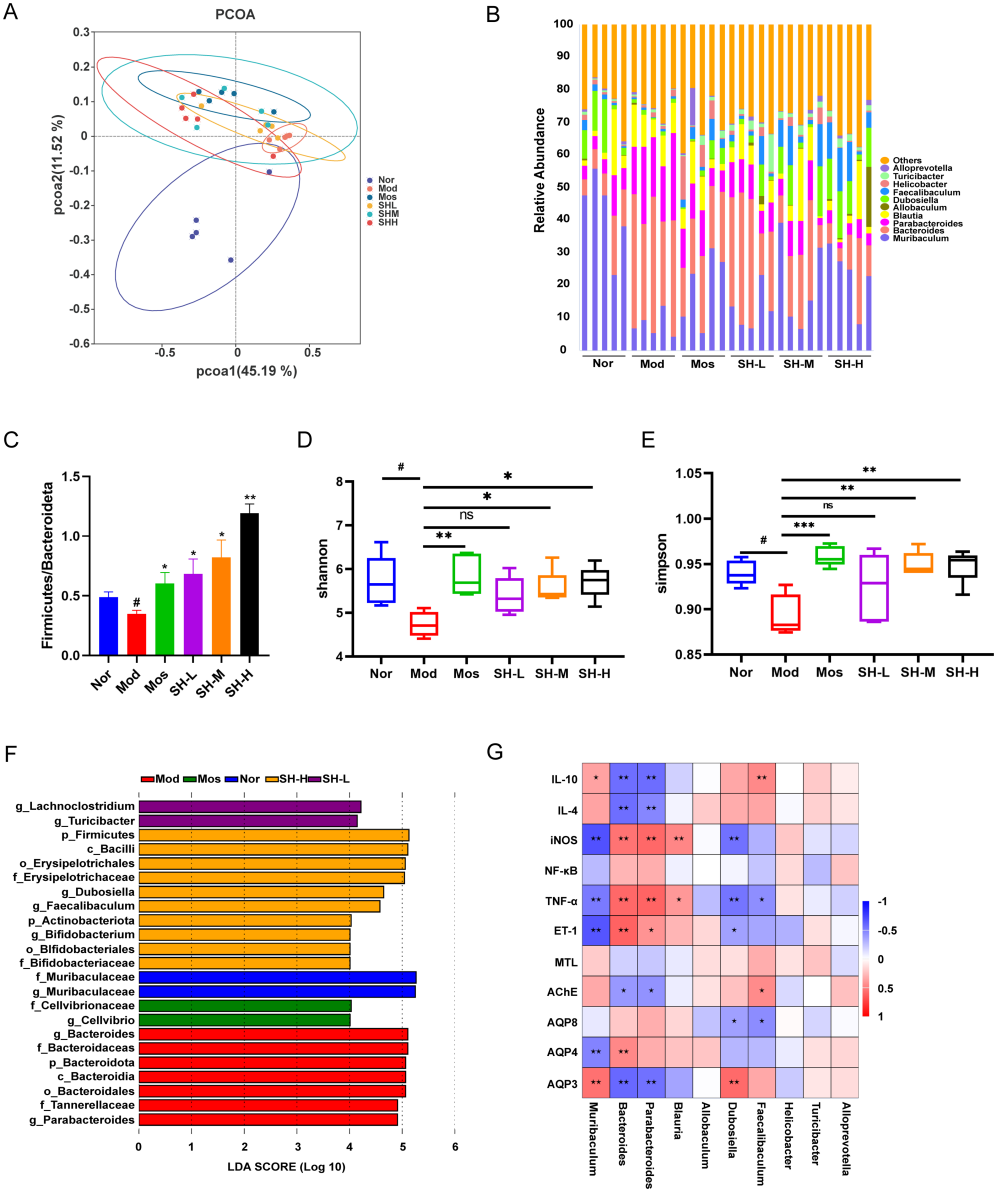
SHTC modulates the gut microbial composition of constipated mice. **(A)** PcoA analysis of gut microbiota in each group; **(B)** Cumulative bar plot of the relative abundance of the top 10 gut microbial species at the genus level; **(C)** Ratio of Firmicutes to Bacteroides at the phylum level; **(D,E)** Alpha diversity analysis with Shannon and Simpson index; **(F)** LEfSe Analysis; **(G)** Correlation analysis between the top 10 genera in abundance at the genus level and environmental factors. Data are reported as mean ± SEM (*n* = 5) and analyzed using One-way ANOVA and Spearman’s rank correlation analysis. ^#^*p* < 0.05, ^##^*p* < 0.01 vs. Normal group; ^*^p < 0.05, ^**^p < 0.01, ^***^p < 0.001 vs. Model group.

A significant body of evidence has highlighted the role of gut microbiota disturbance in the development of constipation. In addition, studies have reported that gut microbiota disorders are a common adverse effect of patients receiving 5-FU treatment (Alexander et al., 2017). For these reasons, we further carried out 16S rRNA analysis to investigate the effect of SHTC on the homeostasis of the gut microbial community. As shown in Figure 3A, in the PcoA analysis, the intestinal flora composition of mice in the model group was markedly different from that of mice in the normal group; the flora composition of the SHTC-treated group was closer to that of the normal group, which indicated that the intestinal flora composition of mice after SHTC treatment was similar to that of the normal group. In addition, the distributions of the bacteria were analyzed. As shown in Figure 3B and Supplementary Figure S3A, which display the top 10 genera in terms of abundance, 5-FU treatment markedly increased the abundance of harmful bacteria, including *Bacteroides* and *Parabacteroides* whereas decreased that of beneficial bacteria, including *Muribaculum*, *Dubosiella*, and *Faecalibaculum*. With the treatment of SHTC, the relative abundance of these bacteria was regulated to levels close to those of the normal group. Notably, these beneficial bacteria are closely associated with increased production of SCFAs. Among them, *Muribaculum* primarily generates acetic acid and small amounts of propionic acid through the fermentation of carbohydrates (Ayimbila et al., 2025). Studies on *Dubosiella* have shown that strains within this genus can promote butyric acid production via specific metabolic pathways (Gao et al., 2024). Additionally, the study found that *Faecalibaculum* can significantly promote the biosynthesis of butyric acid in the colon, which regulates G protein-coupled receptor 109A (GPR109A) to improve ulcerative colitis (Song et al., 2025). Under physiological conditions, the ratio between *Firmicutes* and *Bacteroides* (F/B) is relatively stable, whereas a disruption in their ratio may lead to abnormal conditions such as colitis and constipation (Qi et al., 2023). In our study, we observed that the ratio of F/B was significantly lower in the 5-FU model group compared to the normal group, whereas the ratio of F/B was elevated in both the positive drug and the SHTC administration groups (Figure 3C). The Alpha diversity indices of each fecal sample were also calculated to analyze the diversity and richness of the gut microbiota. In the 5-FU model group, Shannon and Simpson indices were decreased. However, the administration of SHTC increased the Shannon and Simpson index (Figures 3D,E). LEfSe analysis (LDA score>4) indicated that the abundance of pernicious bacteria *Bacteroides* was higher in the model group, while the beneficial bacteria (*Lachnoclostridium*, *Bifidobacteriaceae*, *Faecalibaculum*, *Dubosiella*) were more abundant in SHTC administration group (Figure 3F). Among them, *Lachnoclostridium* has been reported to be a core producer of butyric acid in the gut (He et al., 2025). *Bifidobacterium* could metabolize oligosaccharides into acetate. Enrichment of *Bifidobacterium* significantly elevates gut acetate concentrations, reduces intestinal pH, and thereby suppresses the proliferation of pathogenic bacteria (Ayimbila et al., 2025).

To investigate the correlation between gut microorganisms and environmental factors, we analyzed the correlation between the top 10 bacteria in terms of abundance (genus level) and inflammatory factors, intestinal motility regulatory factors, and AQPs. As shown in Figure 3G, the results revealed that intestinal bacteria with different functions have various relationships with environmental factors. For example, the abundance of *Bacteroides* and *Parabacteroides* was positively correlated with the levels of pro-inflammatory factors but negatively correlated with the levels of anti-inflammatory factors *Il-4* and *Il-10*. Meanwhile, these bacteria have a negative relationship with MTL, AChE, *Aqp4*, and *Aqp8*, while have a positive relationship with ET-1 and *Aqp3*. These observations suggested that SHTC may alleviate intestinal inflammation, alter levels of intestinal motility regulatory biomarkers, and increase intestinal water content by modulating the intestinal microbiota.

### 3.6 Therapeutic effect of SHTC could be induced by microbiota transfer

**Figure 4:**
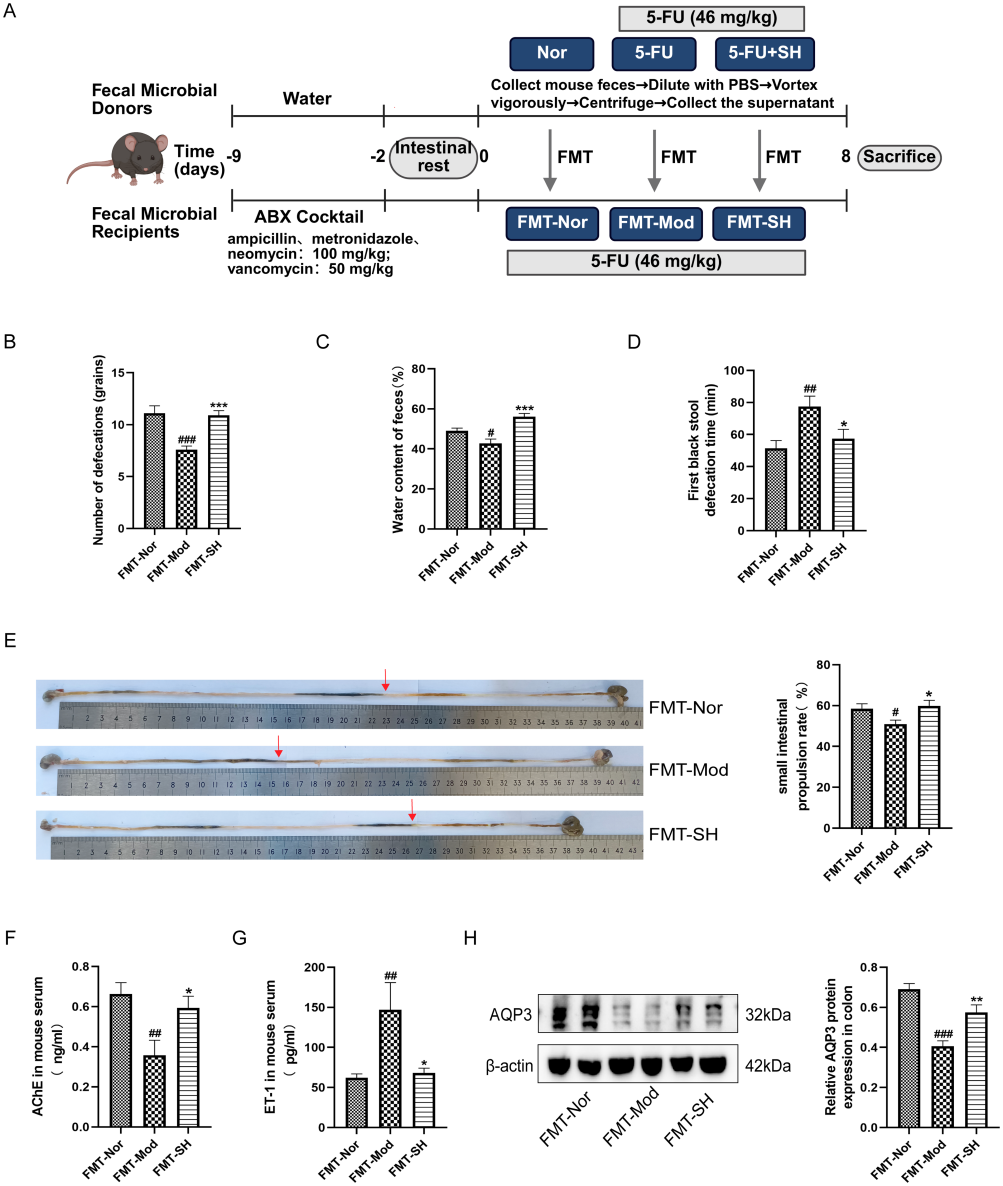
FMT relieves constipation symptoms in mice. **(A)** FMT Experimental Design; **(B)** Number of defecations in recipient mice; **(C)** Fecal water content of recipient mice; **(D)** Time to first black stool in recipient mice; **(E)** Small intestinal propulsion in recipient mice. **(F,G)** Serum levels of AChE and ET-1 in recipient mice; **(H)** Expression of AQP3 protein in colonic tissues of recipient mice. Data are reported as mean ± SEM (*n* = 5/8) and analyzed using One-way ANOVA. ^#^*p* < 0.05, ^##^*p* < 0.01, ^###^*p* < 0.001 vs. FMT-Nor group; ^*^p < 0.05, ^**^p < 0.01, ^***^p < 0.001 vs. FMT-Mod group.

To confirm whether the effect of SHTC on constipation is related to gut microbiota, we further conducted FMT experiments (Figure 4A). The results showed that the mice received microbiota from SHTC-treated mice had no alterations in body weight, food intake, and water intake (Supplementary Figure S4). However, in these mice, the number of defecations, fecal water content, defecation time, and intestinal propulsion rate were restored to a healthy state (Figures 4B–E). The results of ELISA showed that the mice in the FMT-SH group significantly restored the level of AChE (Figure 4F) and decreased the level of ET-1 (Figure 4G). The protein expression of AQP3 in the colonic tissues of recipient mice showed that AQP3 in the mice of the FMT-Mod group was lower than that of the FMT-Nor group. However, the expression of AQP3 was significantly increased in the FMT-SH group (Figure 4H). These results confirmed that the therapeutic effect of SHTC on 5-FU-induced constipation may be related to its ability to regulate the gut microbiota.

### 3.7 Regulatory effect of SHTC on short-chain fatty acid

**Figure 5:**
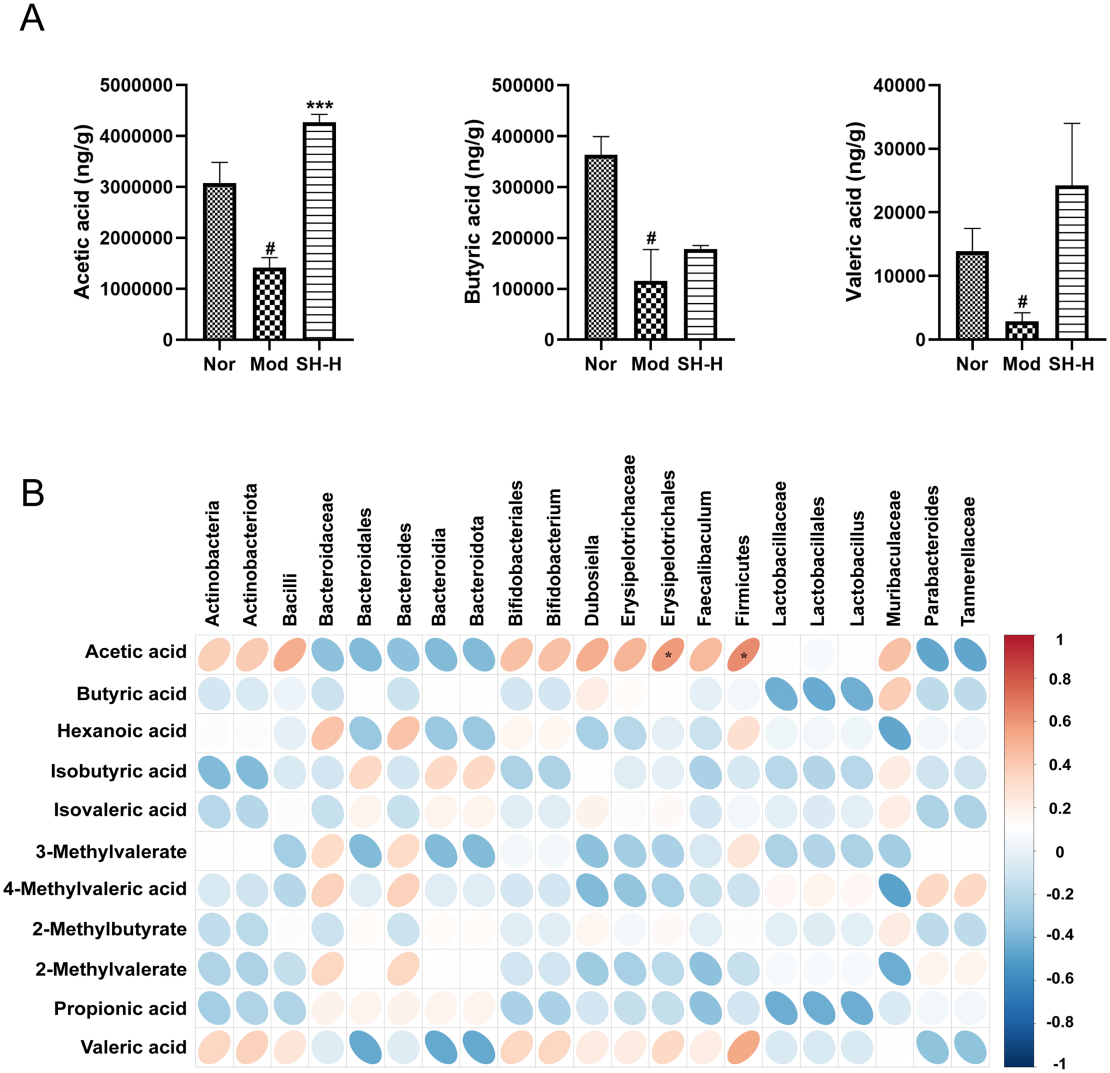
Effect of SHTC on SCFAs content in feces. **(A)** Content of SCFAs in mice feces (ng/g). **(B)** Pearson correlation analysis. Data are reported as mean ± SEM (*n* = 4) and analyzed using One-way ANOVA and Spearman’s rank correlation analysis. ^#^*p* < 0.05 vs. Normal group; ^*^p < 0.05, ^***^p < 0.001 vs. Model group.

SCFAs are major microbial metabolites produced by the gut microbiota through glycolysis. A previous study suggested that the level of SCFAs in the feces is related to the development of constipation (Zhuang et al., 2019). In this study, we used HPLC-MS to detect the content of SCFAs in feces and investigated whether SHTC has a regulatory effect on SCFAs. The results were shown in Figure 5A and S3B, the levels of acetic acid, butyric acid, and valeric acid were significantly decreased in the constipated mice compared with those in the Nor group. The levels of acetic acid, butyric acid, and valeric acid were elevated in the SHTC-treated group, with acetic acid being the most significantly increased one. Moreover, the result of Pearson correlation analysis showed that the level of acetic acid was positively correlated with *Erysipelotrichales* and *Firmicutes* (Figure 5B). The above results suggested that SHTC may play a regulatory role in SCFAs by regulating intestinal bacteria.

### 3.8 Acetic acid increases expression of AQP3 by activating PI3K/AKT pathway

**Figure 6:**
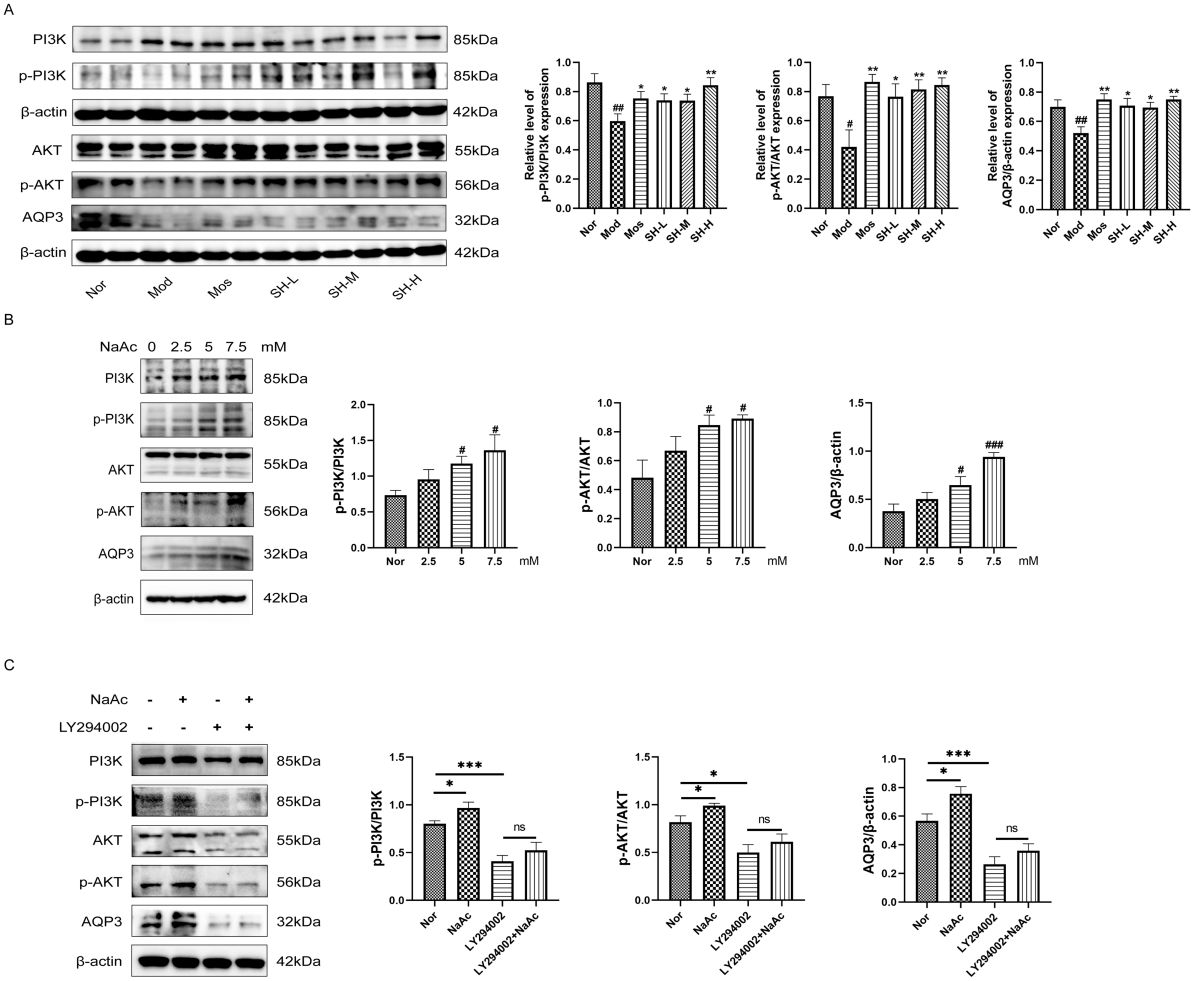
SHTC alleviates constipation in mice by activating the acetate/PI3K/AKT/AQP3 signaling pathway. **(A)** Protein expression levels of PI3K, AKT and AQP3 in the colon; **(B)** Effects of different concentrations of sodium acetate on PI3K/AKT/AQP3. **(C)** The PI3K inhibitor (LY294002) prevented the up-regulation of AQP3 by sodium acetate. Data are reported as mean ± SEM (n = 3/6) and analyzed using One-way ANOVA. ^#^*p* < 0.05, ^##^*p* < 0.01, ^###^*p* < 0.001 vs. Normal group; ^*^p < 0.05, ^**^p < 0.01, ^***^p < 0.001 vs. Model group.

Recent studies have found that SHTC had an activating effect on PI3K/AKT pathway in the liver (Wang et al., 2024). Its main components, naringin and aloin, can also regulate the PI3K/AKT pathway to exert their effects (Lu et al., 2024; He et al., 2021). It has also been shown that activation of PI3K/AKT pathway could promote the expression of AQP3 (Zhang et al., 2022; Park et al., 2022). In addition, considering that SHTC significantly increases the level of acetic acid, we query whether SHTC plays a regulatory role in the activation of PI3K/AKT/AQP3 pathway *via* alternating the production of acetic acid, which further improved 5-FU induced constipation. To find the answer, we first detected the changes in the protein levels of PI3K/AKT and AQP3 in mouse colon tissues, as shown in Figures 6A 5-FU significantly reduced the phosphorylation of PI3K, AKT, and the protein expression of AQP3 in the colon tissues. After treatment with SHTC, the expression of p-PI3K, p-AKT, and AQP3 increased significantly. The dose-dependent upregulation of AQP3 aligns with the action of SHTC on p-PI3K and p-AKT. This association between dosage and functional proteins underscores the necessity for meticulous dose optimization in future studies.

In addition to *in vivo* experiments, we treated IEC-6 cells with sodium acetate to simulate the microenvironment of the mice after SHTC administration. We found that sodium acetate significantly promotes the phosphorylation of PI3K and AKT and the expression of AQP3 in Figure 6B. Furthermore, we exposed the cells to the inhibitors of PI3K. After the experimental treatment, the expression of AQP3 was detected. As shown in Figure 6C, sodium acetate significantly promoted the phosphorylation of PI3K and AKT and up-regulated the expression of AQP3. However, when PI3K was inhibited, sodium acetate treatment did not further upregulate the level of AQP3. These results suggest that SHTC may alleviate 5-FU-induced constipation by increasing acetic acid production and activating PI3K/AKT signaling pathway, thereby promoting the expression of AQP3, improving intestinal water content, and alleviating constipation symptoms.

## 4 Discussion

In this study, we demonstrated that SHTC could effectively improve 5-FU-induced intestinal motility disorders, and the experimental results showed that SHTC was able to increase the water content of feces, regulate the secretion of gastrointestinal-related peptides, promote intestinal peristalsis, and repair the damaged intestinal barrier. In addition, we used FMT experiments to confirm that SHTC improved constipation symptoms by modulating the intestinal microbial structure of constipated mice. Moreover, SHTC was able to increase the content of acetic acid, which activates PI3K/AKT/AQP3 pathway, thereby increasing fecal water content and relieving constipation symptoms.

In this study, we have determined three main components in SHTC, including 2,3,5,4′-tetrahydroxyl diphenylethylene-2-o-glucoside, Naringin, and Aloin. 2,3,5,4′-tetrahydroxyl diphenylethylene-2-o-glucoside is a signature component of *Polygonum multiflorum*. Accumulating evidence has shown that this component could reshape the structure of the intestinal microbiota, specifically by promoting the proliferation of beneficial bacteria such as *Bifidobacterium*, and maintaining the dynamic balance of the *Firmicutes*/*Bacteroidetes* ratio (Bi et al., 2020; Lin P. et al., 2015). Naringin is a flavonoid compound derived from *Citrus aurantium*, which possesses multiple effects including anti-inflammatory, antioxidant, and gastrointestinal motility-promoting properties. Studies have shown that naringin could promote the contractility of rat colonic smooth muscle in a concentration-dependent manner, enhance the intestinal transit rate in rats with gastrointestinal motility disorders, which directly relieve constipation symptoms (Jang et al., 2013; Huang et al., 2018). Additionally, studies have reported that naringin can alter the composition of the intestinal microbiota, increase the relative abundances of *Firmicutes* and *Bifidobacterium* as well as *Lachnospiraceae_bacterium_28-4*, reduce the content of *Proteobacteria*, improve intestinal microbial disorders, and promote intestinal health in mice (Cao et al., 2021; Li X. et al., 2024). Aloin is an anthraquinone component isolated from aloe, demonstrates pronounced laxative effects. Studies have shown that Aloin not only stimulates the intestinal mucosal nerve plexus to promote intestinal peristalsis, but also promotes the secretion of mucin 2 (MUC2) to strengthen intestinal barrier integrity. Concurrently, it significantly suppresses the expression of pro-inflammatory mediators, including TNF-*α*, IL-1β, and myeloperoxidase (MPO) in intestinal tissues (Park et al., 2011; Jiang et al., 2022). Additionally, aloin treatment was able to modulate the composition of the rat intestinal microbiota (Boudreau et al., 2017). Compared with single components, this multi-target and multi-pathway integrated regulation model enables SHTC to exert more comprehensive and mild therapeutic effects, reflecting the unique advantages of traditional Chinese medicine compounds.

5-Fluorouracil is a first-line chemotherapeutic agent commonly used for the treatment of gastrointestinal cancer, breast cancer, and liver cancer. The clinical application of 5-FU has been accompanied by severe gastrointestinal side effects, the most obvious of which are diarrhea and intestinal mucositis. However, several recent pieces of evidence suggested that 5-FU-induced colonic dysmotility outlasts intestinal mucositis (McQuade et al., 2016b; Soares et al., 2008). Animal studies have demonstrated that 5-FU markedly delayed gastric emptying and gastrointestinal transit, as well as reduced fecal excretion (Justino et al., 2015; McQuade et al., 2019). The underlying mechanism involves the following aspects. Firstly, 5-FU treatment can result in a dysregulation of gut microbiota and an imbalance in SCFAs. SCFAs play a vital role in maintaining the health of the colonic epithelium and also have an impact on gut motility. A decrease in SCFAs can lead to diminished colonic contractility and thereby contribute to constipation (Pan et al., 2022; Zhang et al., 2024). Secondly, the damage to the intestinal mucosa induced by 5-FU initiates an inflammatory response. The inflammatory mediators directly act on the enteric nervous system and disrupt the normal neural regulation of gut motility (McQuade et al., 2016a). For these reasons, we used 5-FU (46 mg/kg) every other day for 12 days to establish a mouse model of chemotherapy-induced constipation. By observing and recording the fecal characteristics of the model mice, we found that the mice showed diarrhea on the fourth day of the experiment, and as the experiment progressed, the mice gradually developed constipation. Additionally, we also observed that 5-FU induced gut microbiota dysregulation and intestinal inflammation. Consistent with our observations, a previous study also found that the short-term administration of 5-FU (3 days) increased gastrointestinal transit, while longer-term treatment with 5-FU resulted in delayed gastrointestinal transit (McQuade et al., 2016b). The results showed that short-term treatment with 5-FU accelerated gastrointestinal transit, while longer-term treatment led to delayed gastrointestinal transit and colonic motor dysfunction, thereby inducing constipation symptoms. However, the current study primarily focused on elucidating the mechanism by which SHTC alleviates 5-FU-induced constipation through modulation of gut microbiota and SCFAs metabolism, while the potential neural repair effects on enteric nervous system remained unexplored. In the future study, immunofluorescence will be performed to detect specific neuronal markers (PGP9.5 and HuC/D) in intestinal tissues. Quantitative comparison of enteric neuron density among different groups will be conducted to further elucidate the therapeutic efficacy of SHTC in ameliorating CIC through potential neuroregulatory pathways.

The most well-established murine models for constipation in pharmacological research are the loperamide-induced constipation model and the low-fiber diet-induced constipation model. Loperamide, a selective *μ*-opioid receptor agonist, acts specifically on intestinal μ-opioid receptors. By inhibiting cholinergic neuronal activity within the enteric nervous system, it diminishes acetylcholine release and ultimately reduces intestinal motility (Chen et al., 2012). This model primarily simulates opioid abuse-induced intestinal dysmotility and is suitable for investigating mechanisms and interventions for opioid-induced constipation. The low-fiber diet model reduces fecal bulk and osmotic pressure within the intestinal lumen, thereby weakening mechanical stimulation to the intestinal wall. Simultaneously, it decreases SCFAs production from gut microbial fermentation, further compromising intestinal peristalsis (Li Z. et al., 2024; Yi et al., 2022). This model mimics functional constipation resulting from inappropriate dietary patterns, reflecting the impact of dietary fiber deficiency on intestinal function. Compared to these conventional models, the 5-FU-induced constipation model more faithfully recapitulates the gastrointestinal toxicity of anticancer drugs. It accurately reflects gastrointestinal disturbances encountered by patients during anticancer therapy regimens. For these reasons, the 5-FU induced constipation model is more eligible to this study to evaluate the protective effects of SHTC against chemotherapy-induced gastrointestinal toxicities.

It is well known that anticancer drugs can lead to the disorder of gut microbiota (Touchefeu et al., 2014). The disturbed homeostasis of gut microbiota has been suggested to play a key role in the development of chemotherapy-induced constipation (Hou et al., 2022). Researchers have found that the structure of the intestinal microbiota in patients was markedly changed after undergoing chemotherapy, showing a decrease in *Faecalibacterium* accompanied by an increase in *Escherichia* (Khalif et al., 2005; Montassier et al., 2014). Similar to these observations, in the present study, we found that 5-FU obviously disturbed the composition and *α*-diversity of the gut microbiota in mice, especially decreasing the abundance of beneficial bacteria such as *Muribaculum, Dubosiella*, and *Faecalibaculum*. Administration of SHTC effectively corrected the deregulation of gut microbiota, as indicated by an increase in the abundance of *Faecalibaculum* and a decrease in the abundance of *Bacteroides*. Studies have shown that *Faecalibaculum* is a beneficial bacterium capable of producing SCFAs, and an increase of *Faecalibaculum* can alleviate the constipation symptoms of mice caused by diphenoxylate (Yang et al., 2021). It is worth noting that *Faecalibacterium*, as one of the core genera in the healthy human gut microbiota (Sokol et al., 2008), exhibits significantly higher abundance in the human intestine than in mice (Nguyen et al., 2015). In future clinical practice, it may be explored whether SHTC can enrich *Faecalibacterium* in healthy volunteers or patients with chemotherapy-induced constipation, so as to develop a novel therapy for chemotherapy-induced constipation that targets the elevation of *Faecalibacterium*. Then, in order to confirm that modulation of gut microbiota is a key mechanism by which SHTC relieved constipation, we further performed an FMT experiment. The results demonstrated that the mice receiving the fecal supernatant from SHTC-treated mice have better intestinal propulsion rates and higher fecal water content. These observations suggested that SHTC mediated alteration of gut microbiota is a key factor in the alleviation of constipation.

Moreover, as SCFAs have been shown to play an essential role in maintaining intestinal motility (Martin-Gallausiaux et al., 2021), we further analyzed the concentration of SCFAs in the fecal samples among mice receiving 5-FU and a subsequent SHTC interventional treatment. Our results indicated that the contents of the main SCFAs, such as acetic acid, butyric acids, and valeric acid, were dramatically reduced with the treatment of 5-FU, whereas taking SHTC was valid against these changing trends of SCFAs. Among the SCFAs tested, acetic acid showed the most significant increase in content. It is worth noting that our research findings revealed that beneficial bacterial genera with significantly increased abundances, such as *Muribaculum*, can specifically produce acetic acid through carbohydrate fermentation pathways (Ayimbila et al., 2025). This discovery suggests that SHTC may significantly enhance intestinal acetic acid levels by targeting and regulating the composition of the gut microbiota to promote the enrichment of acetic acid-producing bacterial genera. Studies have reported that acetic acid can accelerate water absorption and stimulate intestinal peristalsis (Smith et al., 2013). Numerous studies have also shown that a close connection between the PI3K/AKT pathway and the pathological mechanisms of constipation. Clinical studies have found that the PI3K/AKT signaling pathway is significantly enriched in the colon tissues of constipation patients compared to healthy control groups (Yao et al., 2024). Furthermore, network pharmacology studies have revealed that the PI3K/AKT signaling pathway serves as a pivotal pathway through which certain natural products exert their laxative effects (Yao et al., 2022). Another study has revealed that activation of PI3K/AKT pathway could significantly increase the expression of AQP3 in the colon, promote water transport into the intestinal lumen, and increase the fecal water content (Park et al., 2022). Given the potential association between the pathogenesis of constipation and the PI3K/AKT pathway, coupled with the regulatory interaction between PI3K/AKT and AQP3, we hypothesize that SHTC may ameliorate 5-FU-induced constipation through modulation of the PI3K/AKT/AQP3 axis. Here, we observed in the colon of mice that PI3K/AKT pathway was obviously activated after the treatment of SHTC, as indicated by the phosphorylation of PI3K and AKT. Meanwhile, the expression of AQP3 was increased in the colon. Additionally, in order to further confirm the regulatory effect of acetic acid on PI3K/AKT/AQP3 pathway, we added acetic acid and PI3K inhibitor to the culture medium of IEC-6 cells. The results showed that when the PI3K signal was blocked, acetic acid could not regulate the expression of AQP3. These results suggested that SHTC increased the level of acetic acid to activate the PI3K/AKT signaling pathway, promote the expression of AQP3, and increase fecal water content. In addition to the PI3K/AKT pathway, classical signaling pathways associated with constipation include the 5-HT signaling pathway (Li D. et al., 2024), SCF/c-Kit signaling pathway (Zhang et al., 2025), and cAMP/PKA signaling pathway (Yu et al., 2024). These pathways play key roles in intestinal motility regulation, secretory function, and neural transmission, with their dysregulation being closely linked to the onset of constipation. As a multi-component herbal medicine, SHTC may possess regulatory potential for these pathways, and future studies can further explore its mechanism of action on these signaling cascades.

## 5 Conclusion

**Figure 7:**
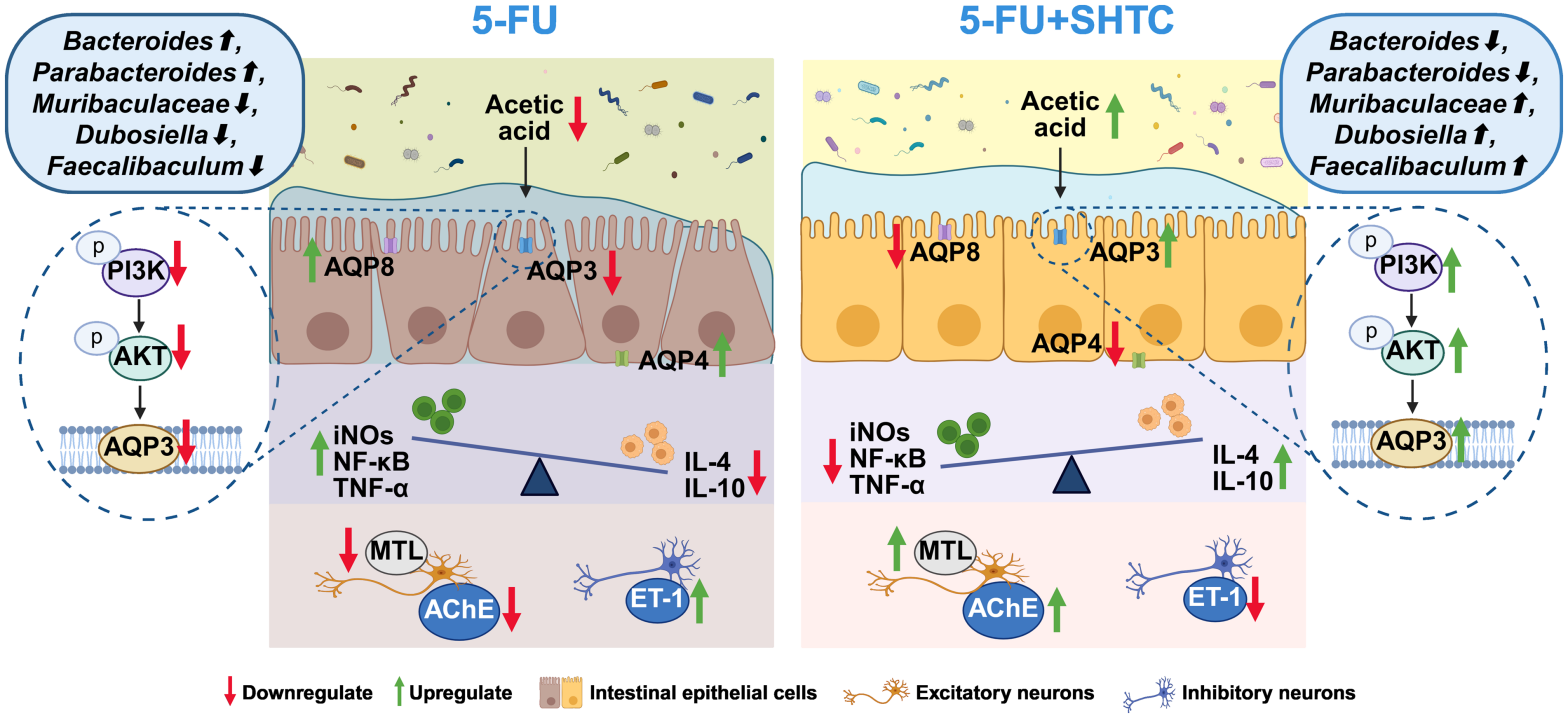
The mechanism of action of SHTC in the treatment of 5-FU-induced constipation in mice. Created in https://BioRender.com.

SHTC is a widely used prescription in the clinical treatment of constipation. In this study, we investigated the efficacy and possible mechanism of SHTC in relieving chemotherapy-induced constipation. The present study innovatively elucidated that the herbal formula SHTC alleviates chemotherapy-induced constipation by concurrently modulating gut microbiota homeostasis and activating the PI3K/AKT/AQP3 signaling pathway (Figure 7). SHTC regulated gut microbiota to enhance acetic acid production, while concurrently employing acetic acid as a signaling mediator to activate PI3K/AKT activity, thereby increasing AQP3-mediated water transport. This multidimensional synergistic mechanism inherent to TCM overcomes the limitations of single-target therapies, such as drug resistance arising from compensatory pathway activation. Therefore, SHTC may be a potential candidate for alleviating chemotherapy-induced constipation. However, there are still some limitations of this study. Although we have proposed a connection between gut microbiota and acetic acid modulation, activation of the PI3K/AKT/AQP3 pathway, and the alleviation of constipation, some of the intermediate steps remain to be further elucidated. For example, the specific strains of gut microbiota that lead to the increase of acetic acid need to be more precisely defined. In future experiments, we will screen strains significantly associated with acetate-producing through metagenomic sequencing analysis, and further validate the ameliorative effects of gut microbiota-derived acetate metabolism on CIC via bacterial colonization assays. Additionally, exogenous acetic acid supplementation experiments are required to directly verify its effect on improving constipation phenotypes. Furthermore, clinical trials need to be conducted to verify the safety and efficacy of SHTC in the treatment of chemotherapy induced constipation, so as to provide support for the clinical application of SHTC.

## Data Availability

The data presented in the study are deposited in the NCBI at the following link: https://www.ncbi.nlm.nih.gov/bioproject/PRJNA1277714, with the accession number PRJNA1277714.
