# Supplementary material for: Shouhui Tongbian Capsule ameliorates 5-fluorouracil induced constipation in mice by modulating gut microbiota and activating PI3K/AKT/AQP3 signaling pathway
Source: Front Microbiol. 2025 Jul 10;16:1596881. doi: 10.3389/fmicb.2025.1596881 (PMC12286977; doi:10.3389/fmicb.2025.1596881)
Supplement: Supplementary file 1 [file Supplementary_file_1.docx]

**Supplementary Materials：**

**Supplementary Tables:**

**Table S1** Sequences of the primers used for real-time PCR analysis.

| **Gene** | **Primer** | **Sequence** |
| --- | --- | --- |
| *Aqp3* | Forward primer | 5’-GCCAAGGTAGGATAGCAAATAA-3’ |
|  | Reverse primer | 5’-TTGAAAACTTGGTCCCTTGC-3’ |
| *Aqp4* | Forward primer | 5’-ACAAGTGCCCGTAATCTGACT-3’ |
|  | Reverse primer | 5’-AATGTCCACACTTACCCCACC-3’ |
| *Aqp8* | Forward primer | 5’-ATCAGCGGTGGACACTTCAA-3’ |
|  | Reverse primer | 5’-CACAGCCAATACCAACAGCAT-3’ |
| *Il-4* | Forward primer | 5’-CTTCCAAGGTGCTTCGCATA-3’ |
|  | Reverse primer | 5’-GATGAATCCAGGCATCGAAA-3’ |
| *Il-10* | Forward primer | 5’-GCTGGACAACATACTGCTAACCGACTC-3’ |
|  | Reverse primer | 5’-CCTTGATTTCTGGGCCATGCTTCTC-3’ |
| *Tnf-α* | Forward primer | 5’-GATGTGGAACTGGCAGAGGAG-3’ |
|  | Reverse primer | 5’-CACGAGCAGGAATGAGAAGAG-3’ |
| *Nf-κb* | Forward primer | 5’-AACACTGCCGACCTCAAGAT-3’ |
|  | Reverse primer | 5’-CATCGGCTTGAGAAAAGGAG-3’ |
| *iNOS* | Forward primer | 5’-TTGGAGCGAGTTGTGGATTGTT-3’ |
|  | Reverse primer | 5’-TAGGTGAGGGCTTGCCTGAGTG-3’ |
| *Gapdh* | Forward primer | 5’-GGTGAAGGTCGGTGTGAACG-3’ |
|  | Reverse primer | 5’-CTCGCTCCTGGAAGATGGTG-3’ |

**Table S2** Linear regression and contents of 2,3,5,4'-tetrahydroxyl diphenylethylene-2-o-glucoside, Naringin, and Aloin. (n = 6).

| **Peak No.** | **Rt (min)** | **Compounds** | **Regression equation** | **R2** | **Content (mg/g)** | **RSD (%)** |
| --- | --- | --- | --- | --- | --- | --- |
| 1 | 4.297 | 2,3,5,4'-tetrahydroxyl diphenylethylene-2-o-glucoside | y = 47,573,484.2542 x + 177,398.7980 | 0.9990 | 11.28 | 4.59% |
| 2 | 6.340 | Naringin | y = 45,593,855.4748 x + 128,161.9660 | 0.9995 | 24.7 | 7.29% |
| 3 | 9.548 | Aloin | y = 51,243,371.4686 x + 9,980.9713 | 0.9999 | 58.74 | 1.57% |

**Table S3** Effect of 5-FU on fecal characteristics of mice at different feeding times.

| Fecal characteristics | Feeding day | Group | |
| --- | --- | --- | --- |
|  |  | Nor | Mod |
| Defecation state | 1 | unobstructed | easy |
|  | 4 | unobstructed | unobstructed |
|  | 8 | unobstructed | a little diffidult |
|  | 12 | unobstructed | hardship |
| Fecal size | 1 | normal | normal |
|  | 4 | normal | large |
|  | 8 | normal | a little small |
|  | 12 | normal | small |
| Amount | 1 | normal | normal |
|  | 4 | normal | normal |
|  | 8 | normal | a little less |
|  | 12 | normal | little |
| Texture | 1 | squishy | a little squishy |
|  | 4 | squishy | diarrhea |
|  | 8 | squishy | a little dry |
|  | 12 | squishy | dry and hard |
| Shape | 1 | oval | oval |
|  | 4 | oval | unformed |
|  | 8 | oval | spherical |
|  | 12 | oval | spherical |

**Supplementary Figures：**

**
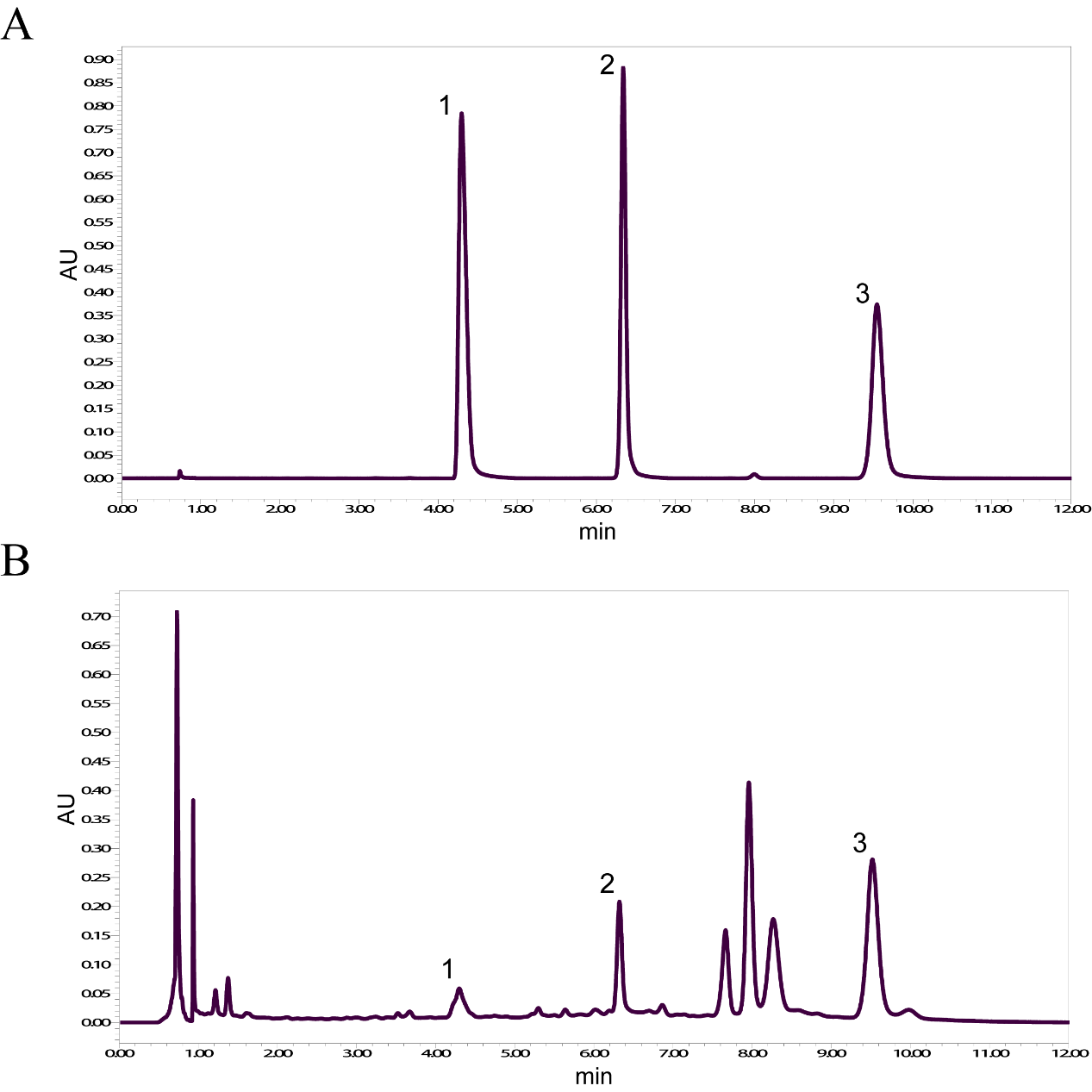
**

**Figure S1:** Identification of active ingredients in SHTC. The typical chromatograms of standard compounds of 220 nm (A) and the sample of 220 nm (B). (1) 2,3,5,4'-tetrahydroxyl diphenylethylene-2-o-glucoside, (2) Naringin, (3) Aloin.


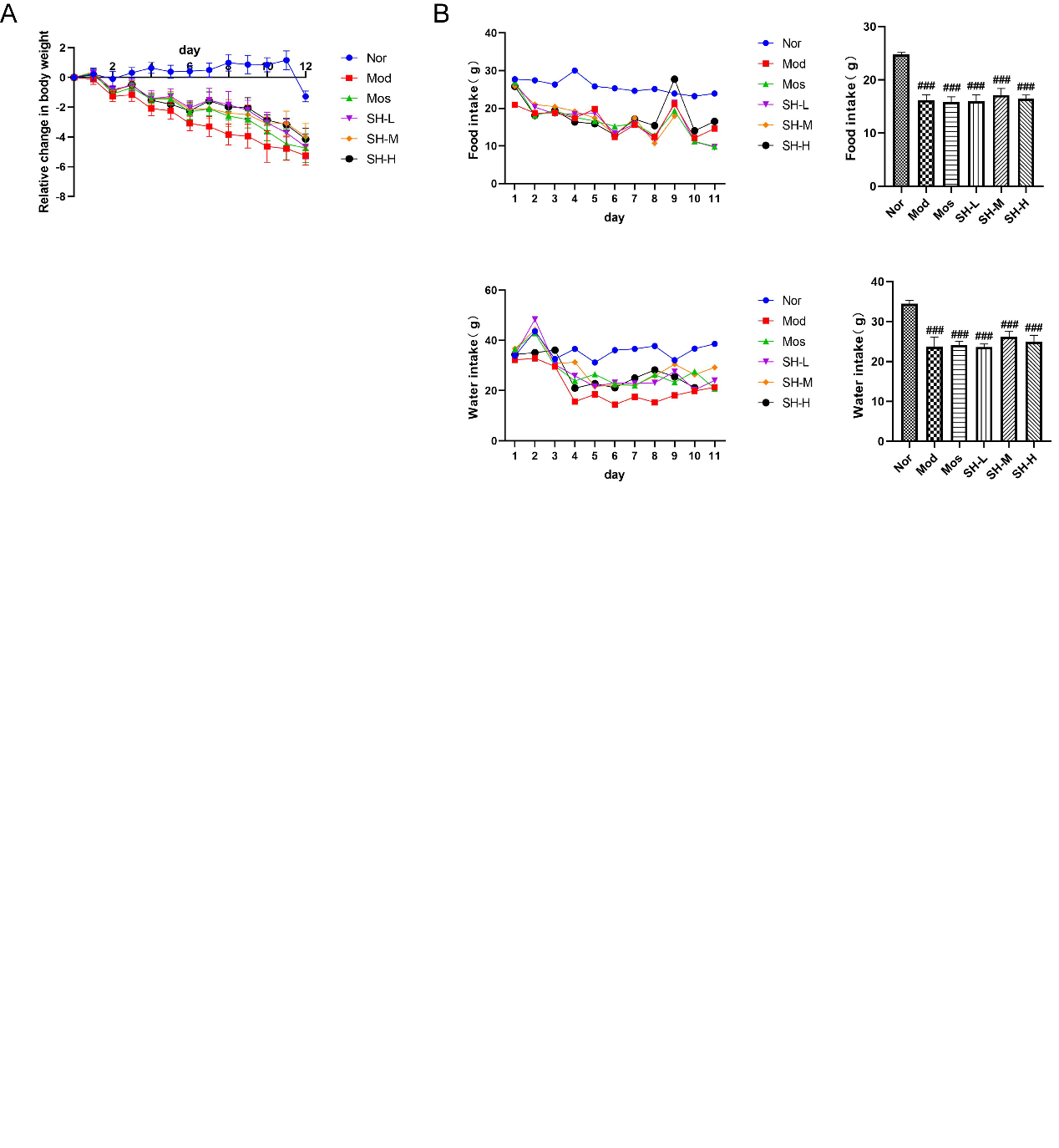


**Figure S2:** Effect of SHTC on body weight and food and water intake in constipated mice. (A) Changes in body weight of mice; (B) Changes in food and water intake of mice. Data are reported as mean ± SEM (n=8). ^###^*p* < 0.001 vs. Normal group.


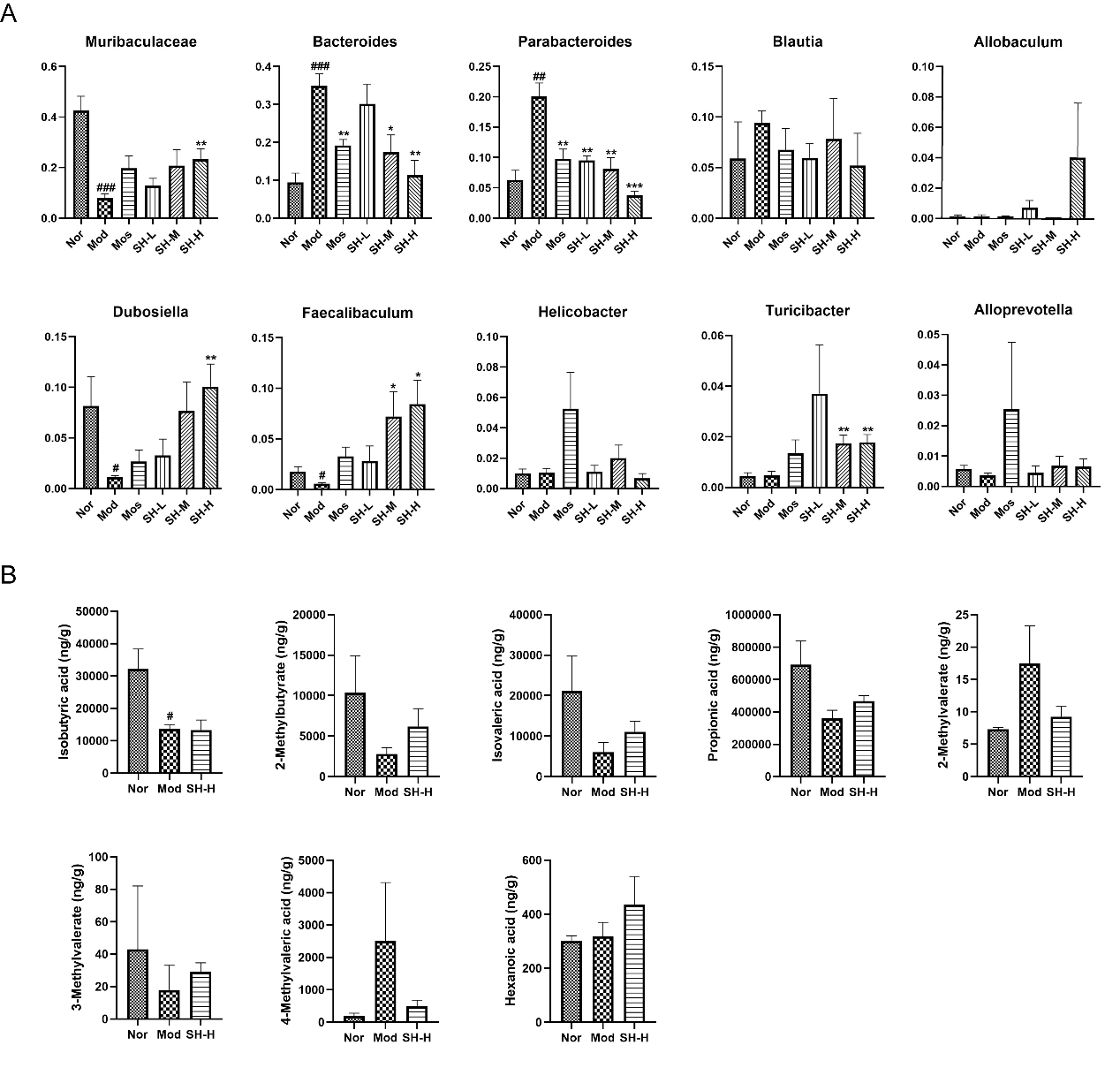


**Figure S3:** Effect of SHTC on the relative abundance of gut microbiota and level of SCFAs. (A) Relative abundance of the top 10 gut microbiota in each group at the genus level; (B) Level of SCFAs in mice feces (ng/g). Data are reported as mean ± SEM (n=4/5). ^#^*p* < 0.05, ^##^*p* < 0.01, ^###^*p* < 0.001 vs. Normal group; ^*^*p* < 0.05, ^**^*p* < 0.01, ^***^*p* < 0.001 vs. Model group.


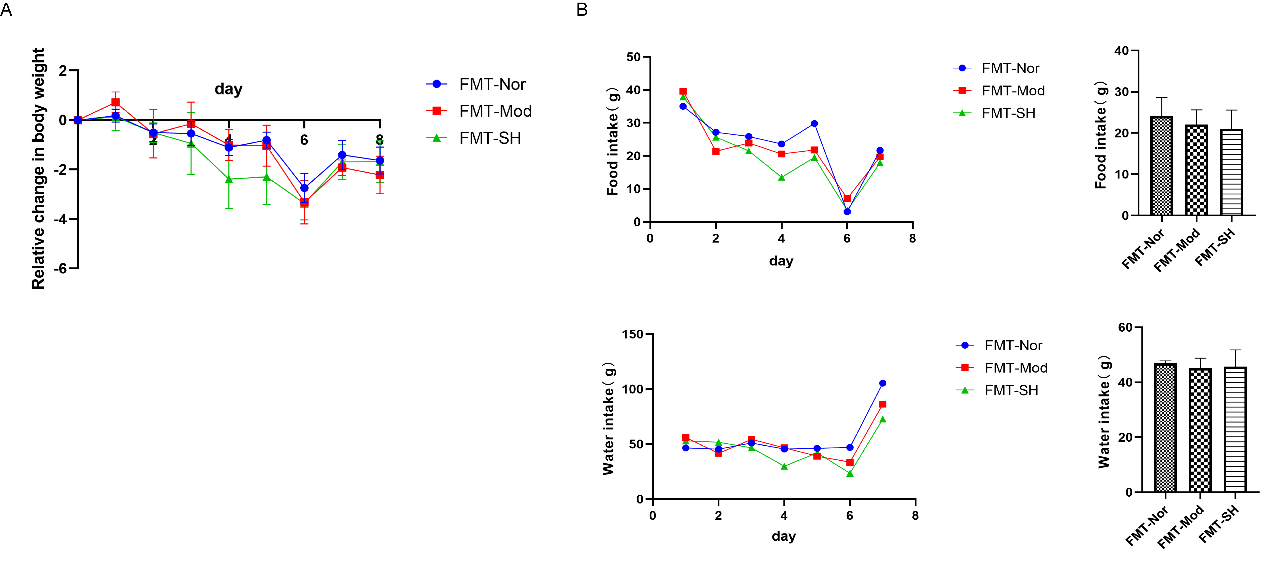


**Figure S4:** Effects of FMT on body weight and food and water intake in constipated mice. (A) Changes in body weight of recipient mice; (B) Changes in food and water intake of recipient mice. Data are reported as mean ± SEM (n=10).
